# Genome Sequence of Singleton Gordonia terrae Bacteriophage Finkle

**DOI:** 10.1128/mra.00693-22

**Published:** 2022-08-25

**Authors:** Seth Ashby, Gavin Bressette, Sydney Brown, Sophie Charles, Melissa Maginnis, Sally D. Molloy, Melody N. Neely

**Affiliations:** a Molecular and Biomedical Sciences, University of Maine, Orono, Maine, USA; b The Honors College, University of Maine, Orono, Maine, USA; DOE Joint Genome Institute

## Abstract

Bacteriophage Finkle is a temperate siphovirus isolated from soil on Gordonia terrae. The 47,895-bp genome has a GC content of 66.6% and encodes 84 protein-coding genes. The genome is not closely related to sequences in the Actinobacteriophage database, sharing less than 35% gene content, and was classified as a singleton.

## ANNOUNCEMENT

Actinobacteriophages (phages) are diverse viruses that infect bacteria of the phylum *Actinobacteria* (1–3). Studying the diversity of phages increases our understanding of phage evolution and is a valuable platform for science education (4). Actinobacteriophage Finkle was isolated from garden soil collected on 01 September 2021 in Brewer, Maine (44.808342 N, 68.729118 W), on the host Gordonia terrae 3612. Soil extracts were prepared in peptone-yeast extract-calcium (PYCa) media and filtered on 0.22-μM filters before inoculating with *G. terrae.* After incubating at 30°C for 2 days, the extract was diluted and plated in soft agar containing *G. terrae* onto PYCa agar. Finkle plaques were purified by three rounds of plaque assays resulting in turbid plaques 1 to 3 mm in diameter on a lawn of *G. terrae* after 2 days incubation at 30°C (5). The Finkle lysate was examined by negative-stained transmission electron microscopy revealing a siphovirus morphology with a long, flexible, noncontractile tail 324 ± 11.5 SE nm in length and an icosahedral head of 62 ± 6.0 SE nm in diameter (*n* = 2).

DNA was extracted from high-titer lysate by phenol-chloroform extraction (6). DNA was prepared for sequencing using the Ultra II library kit (New England BioLabs [NEB], Ipswich, MA) and sequenced on an Illumina MiSeq platform. This process yielded 19,826 single-end 150-bp reads. Newbler v2.9 and Consed v29 were used to assemble the genome sequence and check for completeness, yielding a 47,895-bp genome with 29-fold coverage and a GC content of 66.6%. Genome ends are defined by single-stranded 14-bp 3′ extensions (CTACCTGCGGGGGA). The genome did not share 35% gene content with sequences in the Phamerator Actino_Draft database and was classified as a singleton, which is a phage without close relatives (3, 7, 8).

**FIG 1:**
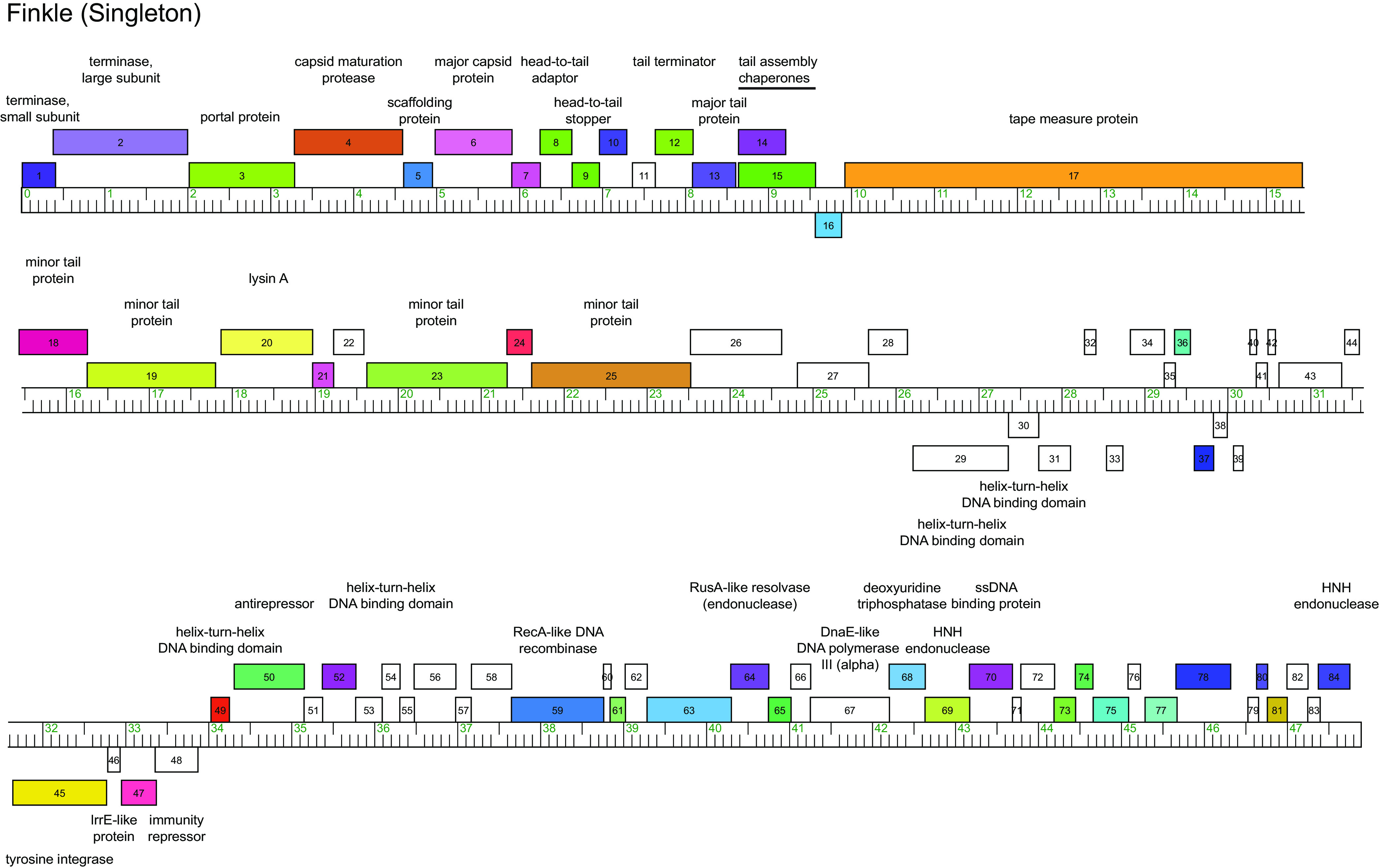
Genome map of *Gordonia* phage Finkle. The genome coordinates are represented by the ruler with kilobase pair units. Forward and reverse genes are represented by colored boxes above and below the ruler, respectively. Genes were assigned to a phamily using Phamerator (7) in the Actino_draft database, and different phamilies are indicated by colors. Genes belonging to Orphams are indicated by white boxes.

Autoannotations of the Finkle genome were performed using GLIMMER v3.02 and GeneMark v2.5 within programs DNA Master v5.23.6 (http://cobamide2.bio.pitt.edu) and PECAAN (https://blog.kbrinsgd.org/) (9, 10). Translational starts were determined manually by identifying starts that include the coding potential predicted in GeneMark.hmm and are conserved across homologs in BLASTp and Starterator (http://phages.wustl.edu/starterator/) analyses (11). Gene functions were predicted using BLASTp, TMHMM, and HHpred (12, 13). No tRNA genes were identified by ARAGORN v1.2.38 and tRNAscan-SE (14, 15). All tools were run with default parameters unless otherwise specified. Comparative genomics was performed using Phamerator in the Actino_Draft database (7). The genome encodes 84 protein-coding genes (Fig. 1). The left arm encodes forward-transcribed structural and assembly genes (gp1 to 25) that belong to gene Phams that is common in mycobacteriophages and *Gordonia* phage clusters (e.g., clusters CZ and P). The right arm encodes forward-transcribed genes (gp49 to 84) including helix-turn-helix DNA binding proteins (gp49 and 54) and a RecA-like DNA recombinase (gp59).

Finkle encodes 37 genes belonging to orphams, which are phams with a single gene member (3). Many of them are located in the center of the genome (23,500 to 34,000 bp) and include both forward- and reverse-transcribed genes. The function of many of these genes is unknown but includes two helix-turn-helix DNA binding domains (gp29 and 30), a tyrosine integrase (gp45), and the immunity repressor (gp48), indicating that Finkle is a temperate phage (16). The immunity repressor is also an orpham; however, homologs were identified within the NCBI database in prophage sequences within bacterial genomes, including Corynebacterium nuruki (GenBank accession no. NZ_CP042429.1), Gordonia iterans (NZ_CP027433.1), and Rhodococcus equi (NZ_LWTY01000001.1).

### Data availability.

Finkle is available at GenBank with the accession no. ON456347 and at the Sequence Read Archive (SRA) with no. SRX14443501.
